# Proof of Concept of an Integrated Laser Irradiation and Thermal/Visible Imaging System for Optimized Photothermal Therapy in Skin Cancer

**DOI:** 10.3390/s25144495

**Published:** 2025-07-19

**Authors:** Diogo Novas, Alessandro Fortes, Pedro Vieira, João M. P. Coelho

**Affiliations:** 1 Departamento de Física, Faculdade de Ciências e Tecnologia, Universidade Nova de Lisboa, Monte da Caparica, 2825-149 Almada, Portugal; d.novas@campus.fct.unl.pt (D.N.); ac.fortes@campus.fct.unl.pt (A.F.); pmv@fct.unl.pt (P.V.); 2 Instituto de Biofísica e Engenharia Biomédica, Faculdade de Ciências, Universidade de Lisboa, Campo Grande, 1749-016 Lisboa, Portugal

**Keywords:** skin cancer treatment, photothermal therapy, laser irradiation system, scanning optics, galvanometric mirrors, thermal imaging, real-time temperature control

## Abstract

Laser energy is widely used as a selective photothermal heating agent in cancer treatment, standing out for not relying on ionizing radiation. However, in vivo tests have highlighted the need to develop irradiation techniques that allow precise control over the illuminated area, adapting it to the tumor size to further minimize damage to surrounding healthy tissue. To address this challenge, a proof of concept based on a laser irradiation system has been designed, enabling control over energy, exposure time, and irradiated area, using galvanometric mirrors. The control software, implemented in Python, employs a set of cameras (visible and infrared) to detect and monitor real-time thermal distributions in the region of interest, transmitting this information to a microcontroller responsible for adjusting the laser power and controlling the scanning process. Image alignment procedures, tunning of the controller’s gain parameters and the impact of the different engineering parameters are illustrated on a dedicated setup. As proof of concept, this approach has demonstrated the ability to irradiate a phantom of black modeling clay within an area of up to 5 cm × 5 cm, from 15 cm away, as well as to monitor and regulate the temperature over time (5 min).

## 1. Introduction

Cancer is a disease characterized by the uncontrolled growth and development of cells in the body due to changes in their genetic code, being considered a global health problem responsible for the death of more than 9.7 million people in 2022 [1]. In the United States alone, the American Cancer Society (ACS) projects that there will be 2 million new cases and about 620,000 deaths in 2025 [2]. According to the World Health Organization (WHO), some of the risk factors for its development include sun exposure, tobacco, alcohol, or even drugs and hormones, to which we can add the aging of the population [3].

Skin cancer is the most common type of cancer worldwide, and its incidence has been increasing significantly [4]. It is generally classified into melanoma and non-melanoma skin cancer. The latter includes basal cell carcinoma (BCC) and squamous cell carcinoma (SCC), which together account for over 99% of non-melanoma cases. The development of skin cancer arises from a combination of genetic and environmental factors, with the most common cause being prolonged exposure to UV light [5,6,7].

Nowadays, there are several options for the treatment of skin cancer, which vary depending on the type and stage of the disease, commonly including surgery (the most common type of treatment), chemotherapy, and radiotherapy. While surgical intervention may face problems related to incomplete tumor removal and high recurrence rates, chemotherapy and radiotherapy have a severe impact on the patient due to their non-specific approach. In this context, phototherapy has gained relevance, especially due to its local action, effectiveness, and reduction of side effects [8,9].

The interaction between laser radiation and tissues has been extensively studied over the years, attracting great interest for both therapeutic and diagnostic purposes. This interaction occurs in different ways and varies from tissue to tissue. The light must be able to penetrate and deliver its energy, depending on the optical absorption properties of the tissue, which are in turn dependent on the wavelength. This dependence is related to the type and number of chromophores (endogenous or exogenous) present and the scattering properties of each tissue, which determine the appropriate laser treatment parameters and protocols to use [10].

The common interaction mechanisms for therapeutic and surgical applications are [11] photochemical reactions (molecules with atoms in an excited state have a higher probability of undergoing chemical reactions with other molecules), photothermal interactions (chromophores, upon absorbing energy, convert it into thermal energy, which can lead to tissue coagulation or even vaporization), photoablation (where electrons, upon absorbing ultraviolet radiation transition from a low-energy orbital to a high-energy non-bonding orbital, causing molecular dissociation), plasma-induced photoablation (an electron, when accelerated by a strong electric field near a laser beam, can collide with a molecule and release another electron, thus triggering a cascade reaction that gives rise to plasma), and photodisruption (mechanical effects that can accompany plasma formation, such as the production of shock waves or cavitation bubbles).

Photothermal therapy (PTT), specifically, uses a laser, often in the near-infrared (NIR) or even deeper infrared region, which, when irradiating the tumor tissue, generates heat, leading to cell death [8]. Studies show that the use of NIR allows for greater beam penetration due to the reduced absorption of these wavelengths by the skin [12]. Also, photothermal agents, such as gold nanoparticles (AuNPs), are commonly used. These nanoparticles accumulate in the tumor and exhibit an absorption peak in those wavelengths, enabling more targeted heating [13,14].

One of the strategies used for PTT involves exposing the tumor region to high temperatures (above 45 °C) for a few minutes; the strong irradiation leads to cell death by thermal ablation [15,16]. Although effective, this strategy is often reserved for situations where the associated risks can be managed, as there is the possibility of causing complications such as hemorrhages, which also limits its combination with other treatments. A second strategy, which offers a safer and more controlled approach, involves setting a temperature between 42–43 °C (mild hyperthermia), which not only promotes cell damage but also increases the permeability of tumor vessels, allowing for greater absorption of photothermal agents [8].

Despite its advantages, PTT still faces limitations that must be addressed for broader clinical application. One of the main challenges is the need for more precise irradiation techniques that allow better control over the illuminated area, adapting to the tumor’s size and shape to minimize exposure of adjacent healthy tissues [17,18]. Additionally, effective temperature regulation is crucial to ensure treatment efficacy while preventing cellular necrosis. Typical methods that can be used to measure the temperature of tissues are [19,20] thermocouples and fiber optic probes (which provide direct, real-time measurements but are intrusive), magnetic resonance thermometry (non-invasive, volumetric temperature mapping and high spatial resolution, but expensive and requiring a complex setup), photoacoustic thermometry (depth-resolved temperature monitoring but requiring complex signal analysis), fluorescent or luminescent nanothermometers (can be targeted to tumor tissue and have potential for high spatial resolution, but often require exogenous agents, are mostly limited to preclinical or small animal models and fluorescence signals may attenuate in deep tissue), and infrared thermography (non-contact, real-time feedback, but only measures surface temperature and are affected by emissivity, ambient conditions, and skin properties).

As a first step to overcome all these challenges, we developed a novel laser irradiation system as a proof of concept targeting future application to skin cancer treatment mediated by photothermal agents. This context, explained in the previous paragraphs, was used to stablish a set of requirements for the system: it should be able to define an irregular area of interest (as most skin tumors are) based on a visible image (assuming that the target is darker and has higher absorption in the NIR than the surroundings due to photothermal agents), establish a temperature to reach (and sustain), scan the area for a defined time and duration, obtain thermal information during irradiation, and provide feedback to the laser system so the defined temperature is maintained. All the parameters should be user defined. With these requirements in mind, the system was designed in a way that could enable control over energy, exposure time, and irradiated area, using galvanometric mirrors and a modulated laser, as well as monitoring and regulating the process temperature with the aid of a thermal and a visible camera. Infrared thermometry was chosen because of its non-contact nature, cost, availability and ease of integration into a portable system. Its integration and testing were performed using a specific phantom that allowed the separation of the evaluation of the engineering parameters of the system from those of the PTT therapy (not addressed for now). Nevertheless, as far as we know, this is an innovative approach for the intended area of application.

In the next sections, the materials and methods used to design, implement and test the system will be presented, followed by the results and corresponding discussion of its application to heating the dedicated phantom. Finaly, we will conclude by summing up the overall system performance and pointing out the necessary work necessary to advance the system toward a medical device supporting PTT.

## 2. Materials and Methods

### 2.1. System Design and Components

#### 2.1.1. Integrated System Overview

**Figure 1:**
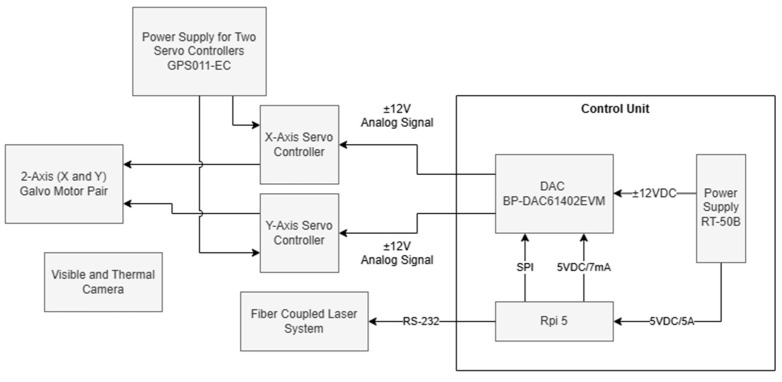
Block diagram of the experimental setup.

The development of this project involved the use of a pair of galvanometric mirrors and their power supply, a fiber-coupled laser, a control system consisting of a microcontroller and a digital-to-analog converter (DAC), and a set of cameras (visible and thermal). The system operates by first detecting the target area to be irradiated, followed by the calculation and execution of the necessary parameters for the precise movement of the mirrors, which are responsible for deflecting the laser. Once the scanning process begins, temperature control is performed through continuous monitoring of the tumor region and constant adjustment of the laser power by the microcontroller. Figure 1 presents a block diagram of the described system configuration that will be detailed in the next sections.

#### 2.1.2. Laser System

The interaction between laser radiation and tissues has been extensively studied over the years given its significant interest for both therapeutic and diagnostic applications. This interaction is highly dependent on the type and concentration of chromophores present, as well as the diffusion properties of each tissue, which determines the appropriate laser parameters and treatment protocols to be used [10]. For PTT, lasers operating in the NIR region of the therapeutic optical window are particularly advantageous, as the low absorption of light by tissues allows for deeper beam penetration [13,14]. Therefore, laser diodes are commonly used due to their lower cost, compact size (facilitating integration), and ability to operate over a wide range of wavelengths, including the aforementioned NIR region [21]. For this study, a Fiber-Coupled Laser System (FC-808–2W, Frankfurt Laser Company, Friedrichsdorf, Germany) was used, emitting at a wavelength of 808 nm and capable of delivering up to 2 W of power, which can be controlled via an RS-232 communication protocol. At the beam output, an FPYL-COL-A collimator (Frankfurt Laser Company, Friedrichsdorf, Germany) was attached, providing a beam diameter of approximately 5 mm with a divergence of 7 ± 1 mrad.

#### 2.1.3. Galvanometric Mirror System

**Figure 2:**
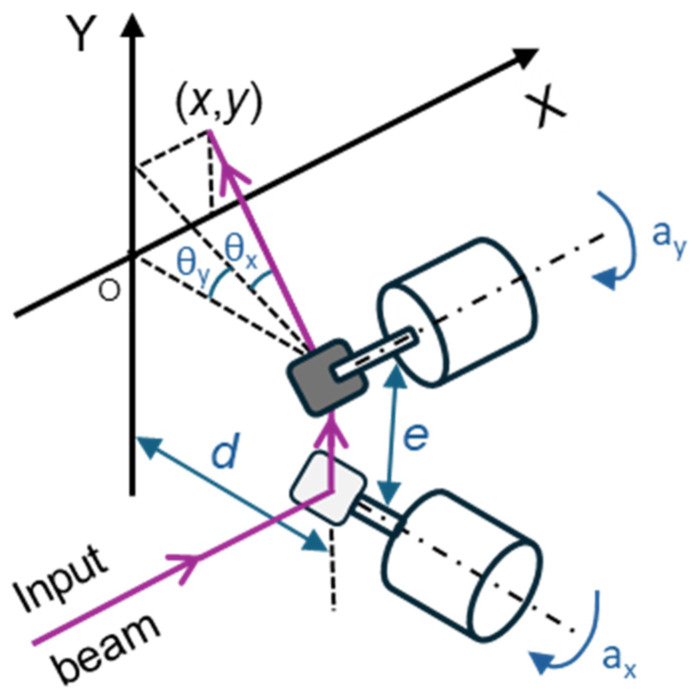
Irradiation system consisting of two galvanometers in a 2D scanning setup, where $\theta_{x}$ and $\theta_{y}$ are the optical scanning angles resulting from each mirror’s rotation; $a_{x}$ and $a_{y}$ are the corresponding mechanical scanning angles; *e* is the offset, corresponding to the distance between the mirrors, and *d* is the distance between the second mirror and the target.

In this study, a large beam diameter dual-axis scanning galvo system (GVS312/M, Thorlabs GmbH, Bergkirchen, Germany) and a GPS011-EC power supply (Thorlabs GmbH, Bergkirchen, Germany) were used. The dual-axis system consists of two mirror and motor assemblies, an X-Y mounting bracket, and two driver cards, which offer the advantage of a simple analog command signal interface. Figure 2 illustrates the system, where $\theta_{x}$ and $\theta_{y}$ represent the optical scanning angles resulting from the mirror rotation, *e* is the offset, and *d* is the distance between the second mirror and the target [22]. Equations (1) and (2) describe the Cartesian coordinates in the plane resulting from the laser deflection by the mirrors [23].

(1) $$x=\left(\sqrt{d^{2}+y^{2}}+e\right)\times \tan\theta_{x}$$

(2) $$y=d\times \tan\theta_{y}$$

#### 2.1.4. Digital-to-Analog Converter and Control Electronics

For the control unit, a Raspberry Pi 5 (Rpi) was selected, featuring a 64-bit Arm Cortex-A76 quad-core processor running at 2.4 GHz. This microcomputer was responsible for hosting and executing the control software and graphical user interface developed for the system, which was implemented using the Flask web framework. However, a drawback of the Rpi is its lack of an analog output, as it only generates digital signals. Therefore, a DAC was required to convert the signal before supplying it to the two driver cards. In this case, a BP-DAC61402EVM (Texas Instruments, Dallas, TX, USA) was chosen—an evaluation module that integrates a 12-bit DAC with two channels, a bipolar output of up to ±20 V, and communication capabilities via a high-speed 4-wire Serial Peripheral Interface (SPI). To power these two components, a 50 W Triple Output Switching Power Supply (RT-50B, MEAN WELL, Fremont, CA, USA) was selected, which provides 5 V, 12 V, and −12 V outputs.

#### 2.1.5. Thermal and Visible Imaging System

The developed imaging system integrates a thermal camera and a visible spectrum camera to monitor and analyze temperature variations during laser-based thermal treatments. The Sunell Dual IP Thermal Cube Camera features a 256 × 192-pixel VOx sensor and a 2 mm lens, optimized for long-wave infrared radiation (LWIR). It measures temperatures between −20 °C and 150 °C with an accuracy of ±2 °C and a thermal sensitivity of ≤65 mK, ensuring the detection of minor temperature variations. Its frame rate enables real-time monitoring, allowing for the dynamic capture of thermal distributions during treatment.

Thermal monitoring is based on the continuous acquisition of infrared radiation emitted by the target surface. The thermal data is extracted from pixel intensity values and processed to generate real-time thermal maps, ensuring precise laser heating control while preventing overheating or insufficient heating, which could compromise the effectiveness of thermotherapy.

### 2.2. Experimental Setup

**Figure 3:**
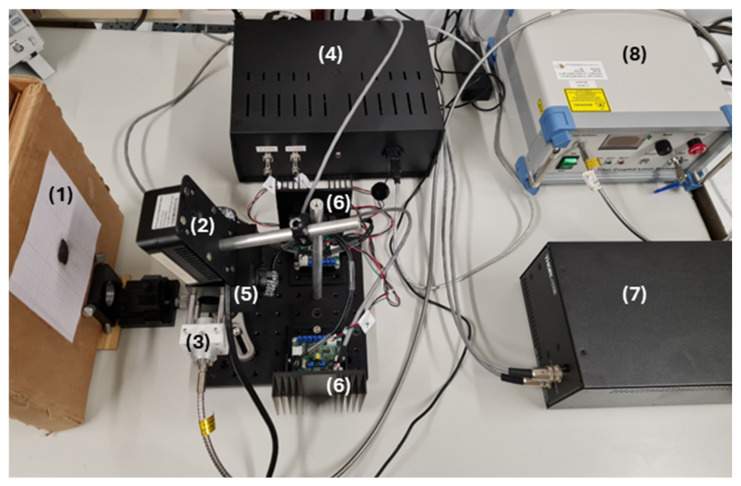
Experimental setup. (1) Target with the sample to be irradiated; (2) Sunell Dual IP Thermal Cube Camera; (3) Collimator (FPYL-COL-A) and 3D-printed component made of PLA filament; (4) Control unit; (5) Dual-axis scanning galvo system (GVS312/M) and (6) driver cards; (7) GPS011-EC power supply; and (8) Fiber-Coupled Laser System (FC-808–2 W).

**Figure 4:**
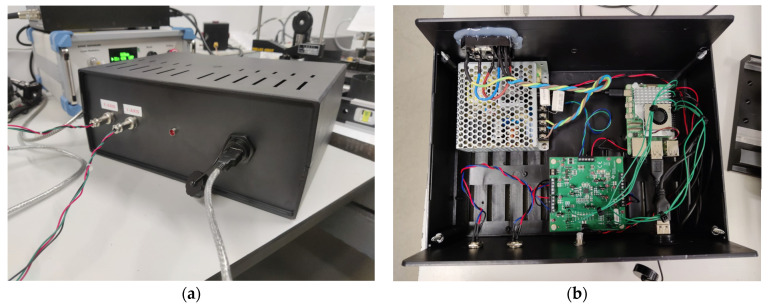
(**a**) Front view of the control unit, showing the two circular 3-pin connectors that link the DAC’s two output channels to the driver cards of the scanning galvo system, an LED, and the USB port that connects the Raspberry Pi to the laser system. (**b**) Top view of the control unit, revealing its interior, where are located, from left to right, the RT-50B power supply, the BP-DAC61402EVM, and the Raspberry Pi 5.

Figure 3 illustrates the experimental setup used in this project. The control unit, consisting of a Raspberry Pi 5, a BP-DAC61402EVM, and an RT-50B power supply, was designed to integrate all components into a single compact module, facilitating connections with the rest of the system (Figure 4). This unit features two signal outputs that connect directly to the driver cards of the scanning galvo system, enabling mirror control, as well as a USB output that establishes communication between the Rpi and the laser system. This allows for real-time power adjustment via RS-232 protocol communication. For beam alignment, a custom component was designed and 3D-printed to center the collimator with the optical system’s input aperture, using assembly rods for fixation. Finally, a target with the sample to be irradiated was positioned 15 cm away from both the camera and the mirrors, maintaining this distance consistently throughout the project. Black modeling clay was selected as a material due to its ease of use, allowing the development efforts to focus primarily on the engineering components. Nevertheless, this material is commonly used as a positive control in PTT tests due to its high absorption in the NIR range [24,25], and regarding our case, added the advantage of being easily modeled to irregular shapes (as those of skin tumors). One should note that there was not a concern to realistically mimic a real biological material due to the high number of external parameters this would add. Thus, the motivation was mainly to choose a highly absorptive material (in the wavelength being considered) capable of being molded into irregular shapes. However, its thermal and optical characteristics differ from those of tissues, even considering that in the PTT of cancer, highly absorbing nanoparticles are incorporated into the tumoral tissue. Also, the lack of information regarding the thermophysical properties of the specific clay used as a target limits a deeper analysis of its effective response to the radiation. Regarding its emissivity, it was considered that it was 0.95 (as that of opaque plastic) while the value for the human skin can be considered around 0.98 [26]. Other important inputs for the operation of the infrared camera are the reflected temperature and ambient humidity, which were considered as 23 °C and 63%, respectively (laboratory established conditions during the period of the tests). Under different conditions (like a more realistic phantom), all of these parameters can be changed.

### 2.3. Software and Control Platform

**Figure 5:**
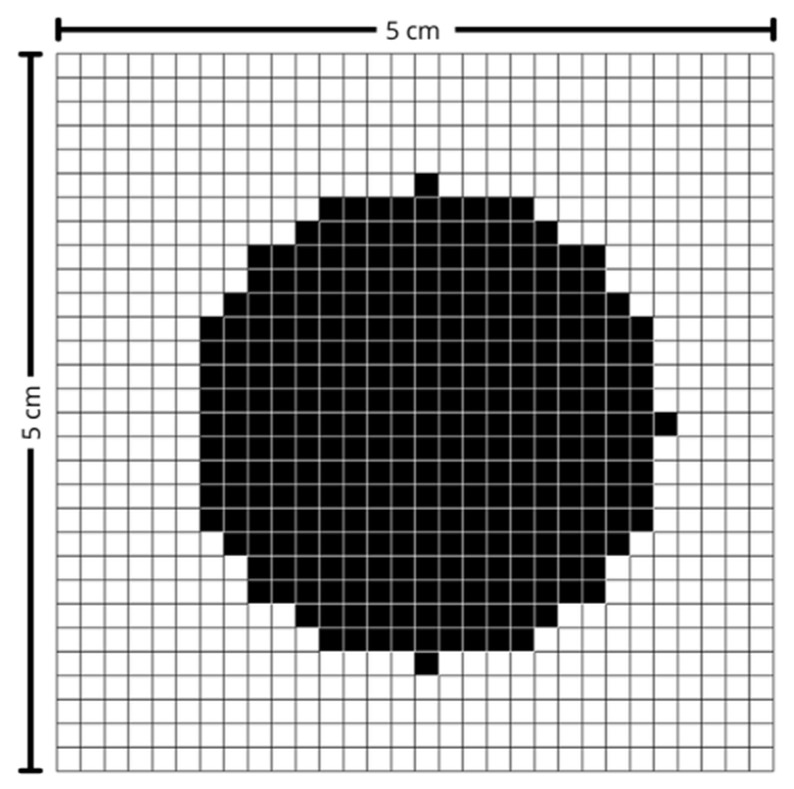
Scanning matrix. Black-marked points indicate the region of interest.

The control software was developed in Python (version 3.13.2) and receives as input a 30-row by 30-column matrix, representing the predefined maximum scanning area. In this matrix, black points indicate the region of interest to be scanned, as illustrated in Figure 5. The matrix size was determined based on the maximum scanning area and the characteristics of the laser used, ensuring the most uniform exposure possible throughout the scanning process.

Melanoma and non-melanoma lesions can, in more advanced stages, reach several centimeters in diameter. Thus, to cover the vast majority of cases, a maximum area of 5 cm in width by 5 cm in height was defined, corresponding to an effective pixel size of approximately 1.67 mm × 1.67 mm. This value was also established considering that the larger the area, the greater the amplitude of movement required by the mirrors, which consequently leads to a reduction in the maximum scanning speed. The laser used has a beam diameter of approximately 5 mm and, for measurement purposes, it was assumed to have a Gaussian profile.

For the correct positioning of the mirrors, the Cartesian coordinates of each point of interest are first calculated (Equations (3) and (4)), where $n_{r}$ represents the row number in the matrix, $n_{c}$ the column number, and $d_{p}$ the distance between two consecutive points.

(3) $$x=\left(n_{c}-15.5\right)\times d_{p}$$

(4) $$y=\left(-n_{r}+15.5\right)\times d_{p}$$

Next, Equations (1) and (2) are used to calculate the optical scanning angles, $\theta_{x}$ and $\theta_{y}$. The scanning galvo system used operates with a scale factor, *s*, of 0.5 V/°, meaning that the voltage supplied by the DAC must follow $V=s\times \theta_{m}$, where $\theta_{m}$ is the mechanical scanning angle.

Furthermore, to ensure that the DAC provides the correct output voltage over time, the microcontroller must send the appropriate digital signal via SPI communication. This signal, expressed in bits, follows Equation (5), where $V_{min}$ and $V_{max}$ are the minimum and maximum output voltages, *N* is the number of bits, and *D* is the computed digital value.

(5) $$D=\frac{V-V_{min}}{V_{max}-V_{min}}\times \left(2^{N}-1\right)$$

### 2.4. Scanning Pattern and System Monitoring

#### 2.4.1. Raster Scan

**Figure 6:**
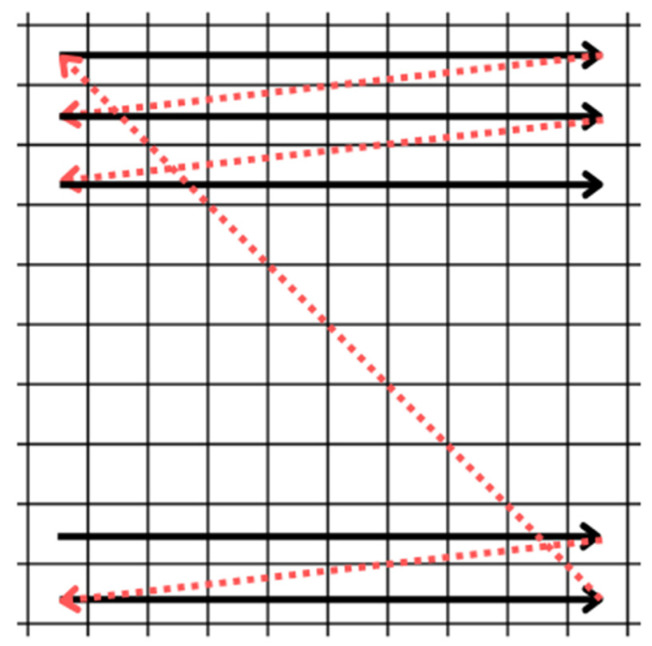
Raster scan pattern. The movement occurs from left to right and from top to bottom. The horizontal and vertical retraces are shown in red.

**Figure 7:**
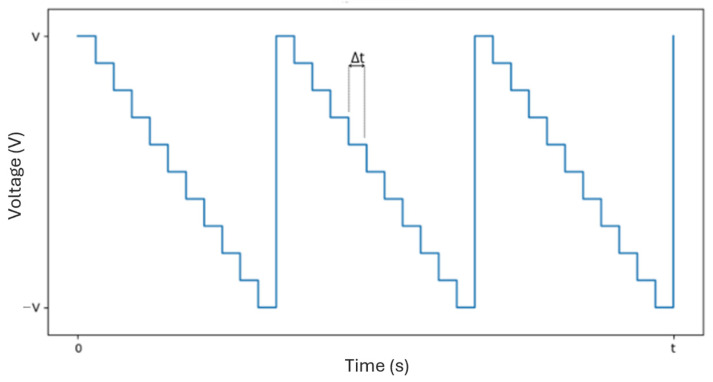
Example of a possible signal for horizontal or vertical scanning (raster scan pattern), with Δt representing the time between voltage updates.

For this work, a raster scan pattern was chosen, as shown in Figure 6. This scanning method was selected due to its simplicity and the uniform exposure it provides across the entire scanning area. While other scanning patterns—such as those based on sinusoidal signals—can offer performance advantages under certain conditions, they are typically more complex to implement and often result in non-uniform coverage. In contrast, the raster scan ensures a consistent and predictable motion, with the beam moving line by line, from left to right and from top to bottom. Whenever the beam reaches the end of a line, it returns to the beginning of the next one (horizontal retrace). Upon reaching the end of the last line, it quickly returns to the upper-left corner to start a new cycle (vertical retrace). Figure 7 illustrates the signal corresponding to this pattern, characterized by small steps due to the process of converting a digital signal into an analog one. Each voltage value moves the beam to the center of a new pixel, and the time between voltage updates, Δt, defines the scanning speed. This approach was chosen because it requires lower temporal precision from the microcontroller and less computational capacity. The signals that control horizontal and vertical scanning are similar in form (approximately a sinusoid), with the horizontal signal having a higher frequency due to the nature of the line-by-line movement of the raster scan. The specific frequencies in each direction depend on the scanning parameters used.

#### 2.4.2. Scanning Times

Controlling the scan time is crucial to ensure a uniform distribution of laser energy across the entire tumor region.

The scanning must be performed fast enough for the exposure to approximate continuous irradiation across the surface, allowing the entire area to heat uniformly. If the scanning is too slow, localized heating occurs, as the laser spends more time on specific regions, resulting in the formation of hot and cold spots due to insufficient temporal overlap of the exposure. On the other hand, excessively high scanning speeds can lead to distortions in the scanning pattern, with the laser beam failing to adequately cover the intended area.

**Figure 8:**
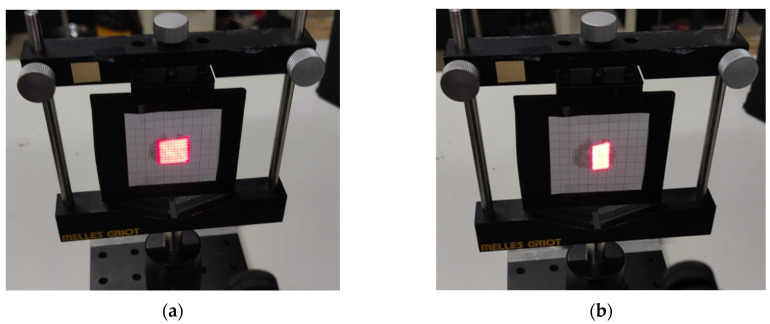
Scanning images with (**a**) moderate scanning time of 0.9 s (no distortion) and (**b**) very short scanning time of 0.25 s (with distortion).

This deformation is caused by the inability of the galvo mirrors to complete the horizontal retrace of the beam within the required time, as illustrated in Figure 8. To address this issue and fully leverage the system’s capabilities, diagnostic outputs available on the servo amplifier boards are used. The position output provides a scaled, real-time signal corresponding to the galvo motor’s instantaneous position. This signal is buffered through the servo amplifier and indicates the actual spatial position of the galvo.

By monitoring both the command signal sent to the servo amplifier and the signal representing the actual position, it becomes possible to detect signs of instability or situations in which the system can no longer achieve the desired scan angles.

### 2.5. System Calibration and Alignment

**Figure 9:**
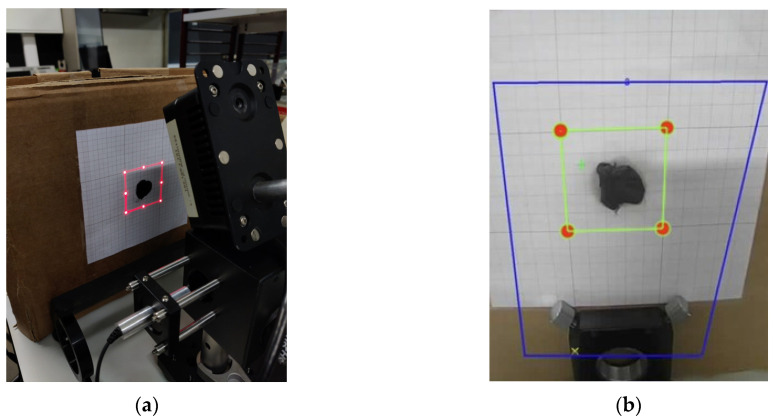
(**a**) Scanning of the maximum defined area perimeter and its adjustment using a standard measurement drawn on graph paper on the target and (**b**) definition of the region of interest in the graphical interface.

System calibration is an essential process to ensure the accuracy and reliability of the scanning procedure, guaranteeing an exact match between the laser projection and the area of interest captured by the camera. This procedure is carried out in two fundamental steps (Figure 9):

1. Laser System Calibration—Using the previously mentioned equations, the positions of eight points along the perimeter of the maximum scanning area at a given distance are calculated. Based on these values, the system projects a luminous square pattern that must align with a reference square drawn on graph paper. The target’s position is adjusted until the projection is aligned.
2. Camera Calibration—Next, a region of interest (ROI) is defined within the camera software interface, corresponding to the area previously aligned by the laser. This adjustment ensures that the captured thermal images are correctly superimposed on the scanning region.

With both steps completed, a precise alignment between the laser and the camera is ensured, minimizing errors and optimizing thermal analysis for monitoring specific targets.

### 2.6. Visible Image Processing

The image acquisition process in the developed system involves the simultaneous capture of both visible and thermal images, enabling a detailed analysis of the thermal distribution within the region of interest. The visible image is obtained through an IP camera by issuing an HTTP GET request to its dedicated endpoint. The function responsible for this acquisition establishes communication with the camera, providing parameters such as image quality and device identifier. Upon receiving a successful response, the image is locally stored in blocks, optimizing data transfer and minimizing potential data loss.

Conversely, the acquisition of thermal images follows a distinct procedure, whereby a specific request is sent to the device to retrieve the infrared image. In parallel, an additional query is executed to extract temperature data corresponding to predefined areas within the camera’s field of view, since it is not possible to access the temperature of individual pixels directly. Parameters such as the target area and the intended action are specified to ensure accurate extraction of the relevant thermal values. The thermal information, collected in real time, is then processed and analyzed, allowing its integration into the system and supporting informed decision-making within the scope of the developed application.

#### 2.6.1. Filters and Threshold

**Figure 10:**
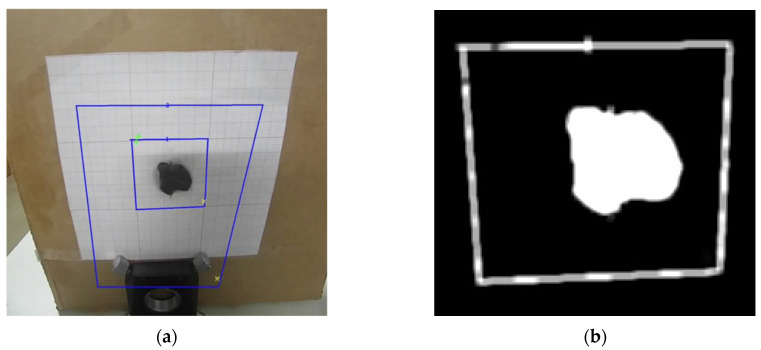
(**a**) Visible image and (**b**) segmented object of study. Blue Boxes represent the regions of interest (central and background), and the colored marks indicate the pixels with the highest (green) and lowest (yellow) temperatures, for each region.

Image processing plays a crucial role in the segmentation and thermal monitoring of the target object, as illustrated in Figure 10. Following acquisition, the images were converted to grayscale, providing a uniform representation of intensity values suitable for the subsequent analysis. This conversion ensures a more consistent representation of visual information, facilitating the identification of regions with greater thermal relevance. Segmentation was carried out using an inverse binary threshold, which highlighted lower intensity areas—typically associated with zones of higher thermal absorption—allowing precise isolation of the target region.

To enhance segmentation quality, morphological operations were applied, particularly the closing technique, which removed noise and filled small internal gaps in the segmented regions. In addition, a smoothing filter was used to attenuate abrupt intensity variations and reinforce relevant contours. These techniques ensured the structural consistency of the region of interest, thereby improving the reliability of subsequent phases of analysis and thermal control.

#### 2.6.2. Keystone Distortion

**Figure 11:**
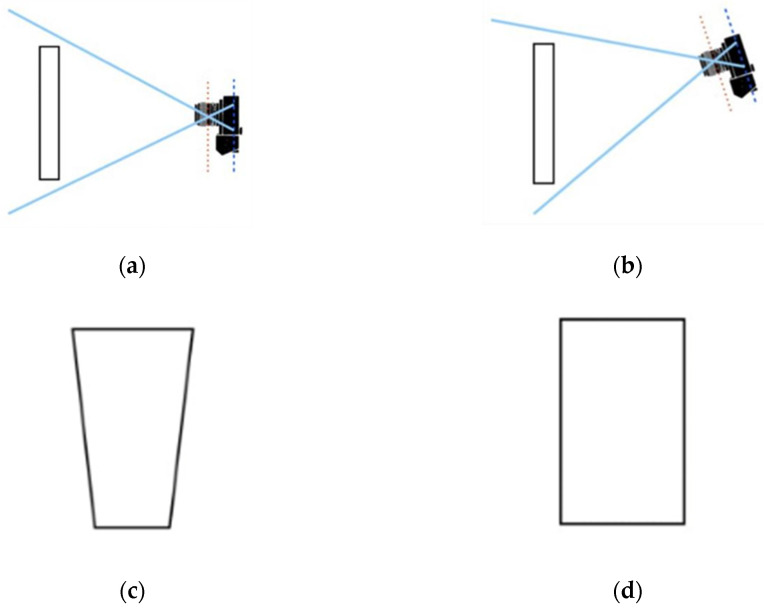
Views with (**a**) the camera perpendicular to the imaged plane, and (**b**) the camera tilted relative to the imaged plane. (**c**) Illustration of the Keystone Effect, and (**d**) resulting image after correction. Adapted from [28].

The inclination of the camera to the target, as illustrated in Figure 11, introduces a geometric distortion known as the Keystone Effect, which alters the true shape of the captured image. To correct this deformation, the image processing workflow incorporates three main steps: contour detection, identification of the region of interest vertices, and application of a perspective transformation [27,28].

**Figure 12:**
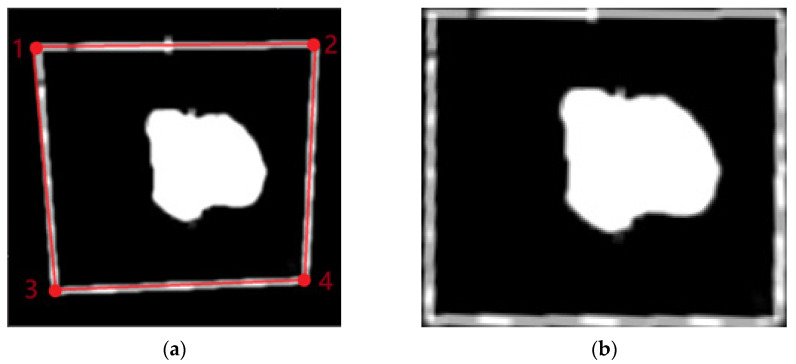
(**a**) Maximum contour of the scan area identified in red and (**b**) image view. The numbers in red represent the four corner points ordered to ensure the correct spatial correspondence.

First, the contour corresponding to the maximum scanning area in the segmented image is identified. Then, four corner points representing the vertices of a quadrilateral are extracted from this contour and logically ordered to ensure the correct spatial correspondence (Figure 12).

Finally, a perspective transformation is applied to adjust the image so that the edges become parallel and proportionate, effectively correcting the distortion caused by the camera tilt. This step is essential to ensure geometric accuracy in the analyzed area, enabling reliable measurements and consistent alignment with the scanning system.

#### 2.6.3. Image Transformation

After the perspective transformation, the image is cropped to isolate the region of interest and resized to fixed dimensions of 30 × 30 pixels using OpenCV’s cv2.resize function with area interpolation. This resizing standardizes the size of the image for subsequent analyses, such as threshold application and binary conversion.

**Figure 13:**
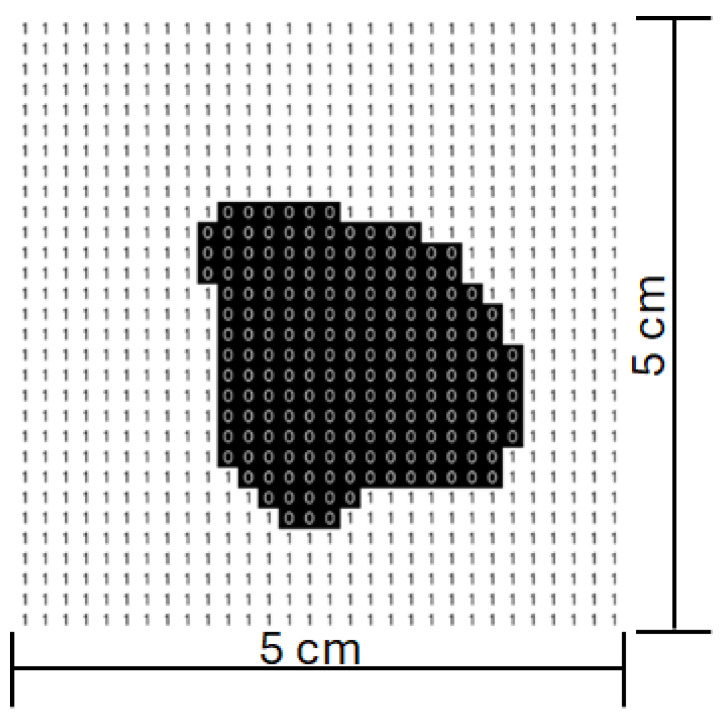
Scanning matrix after image processing.

The final image undergoes morphological operations, such as opening with a kernel, to remove noise, and is then converted into a binary matrix representing values of 0 and 1, as shown in Figure 13. This compact and standardized binary matrix is prepared for transmission in JSON (JavaScript Object Notation) format, ensuring compatibility for integration with external systems.

### 2.7. Thermal Image Processing

For the thermal image, segmentation is performed using a binary criterion that distinguishes regions of interest based on intensity differences. To enhance the definition of contours and eliminate unwanted interference, a morphological operation combining dilation and erosion is applied. This operation helps to uniformize the segmented areas, strengthening the removal of noise and improving the accuracy of relevant structure detection. Geometric distortion follows the same process mentioned earlier, involving a transformation that adjusts the image perspective to ensure consistent alignment of the edges. This process is based on the reorganization of key points extracted from the contours, enabling the analyzed area to be converted into a regular, uniform format, essential for the reliability of the extracted thermal data.

Furthermore, to compensate for discrepancies in the overlap between visible and thermal images, a spatial alignment mechanism is implemented, using reference coordinates to adjust the correspondence between both domains. This refinement ensures that thermal information is properly integrated with the reference image, allowing for accurate analysis in systems utilizing multiple sensors.

#### 2.7.1. Correction of the Parallax Effect

Parallax refers to the apparent displacement of an object when observed from two distinct viewpoints, a phenomenon that, in the present system, arises due to the spatial arrangement of the cameras, with the thermal camera positioned a few centimeters below the visible camera. This offset introduces discrepancies between the images captured by each device, making it necessary to apply dedicated correction techniques to ensure spatial coherence and analytical accuracy.

**Figure 14:**
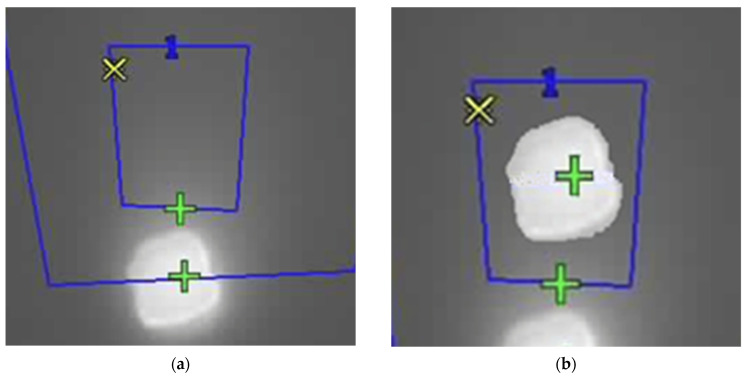
Thermal images (**a**) with the parallax effect and (**b**) with its correction. Blue Boxes represent the regions of interest (central, with a blue “1”, and background), and the colored marks indicate the pixels with the highest (green) and lowest (yellow) temperatures, for each region (yellow marks are superposed in the images).

Parallax correction in thermal images is performed through a structured set of image processing techniques, ensuring precise alignment between regions of interest in both visible and thermal images. Initially, both images are rescaled to the same dimensions and converted to grayscale. A binary threshold is then applied to enable effective segmentation of the relevant areas. To enhance the definition of the identified structures and eliminate undesired noise, morphological operations are applied to refine contours, ensuring greater precision in the detection of the elements to be aligned. Subsequently, the regions of interest are identified and isolated, and their respective centroids are calculated based on the contour distribution. These reference points are essential for determining the current coordinates and the target points used to compensate for the misalignment caused by the parallax effect. The correction process involves applying a spatial adjustment that isolates and shifts the region of interest within the thermal image, ensuring its exact overlap with the corresponding area in the visible image, as illustrated in Figure 14. The full process is properly explained in [29]. During this procedure, checks are performed to ensure that the adjusted region remains within the image boundaries, thus preventing distortions or loss of information.

#### 2.7.2. Thermogram

**Figure 15:**
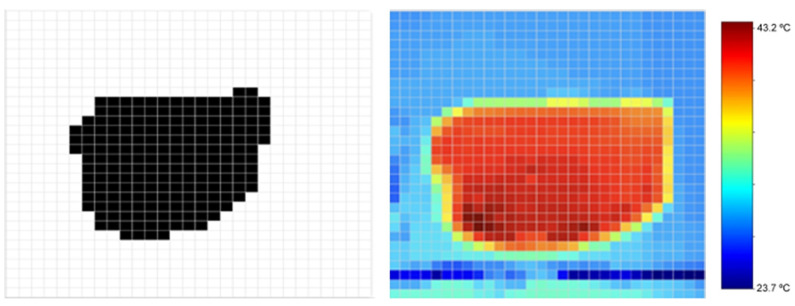
Digital image of the study object (**left**), heat map obtained using the thermal image and temperature parameters (**center**), and corresponding color bar with temperature range (**right**).

The conversion of thermal image intensity values into temperature data is carried out through a normalization and scaling process, ensuring accuracy in the interpretation of the recorded thermal variations. The camara produces a thermal image of 256 × 292 pixels with a temperature accuracy of ±2 °C and an operational range of −20 °C to 150 °C. The colormap of the image was set to be grayscale, with the intensity in each pixel proportional to its temperature. The camera also provides, for each image, the maximum (lighter pixel) and minimum (darker pixel) temperature measured. With this information it is possible to build an image, where each pixel is the value of the temperature, using Equation (6). Thus, each pixel is translated into its corresponding real temperature, resulting in a high-precision thermal matrix that preserves the original resolution of the image (Figure 15).

(6) $$T_{pixel}=\frac{\left(T_{max}-T_{min}\right)}{\left(I_{max}-I_{min}\right)}\left(I_{pixel}-I_{min}\right)+T_{min}$$

where $T_{pixel}$ is the temperature of the calculated pixel; $T_{max}\;and\;T_{min}$ are the maximum and minimum temperature of the image, respectively; $I_{pixel}$ is the intensity of the pixel; and $I_{max}\;\mathrm{and}\;I_{min}$ are the maximum and minimum pixel intensity of the image, respectively.

This calculation plays a crucial role in extracting reliable thermal information from the captured image, enabling a detailed analysis of temperature fluctuations in a specific area. The obtained thermal matrix can later be refined for discrete representation or used in subsequent system stages, such as the creation of heat maps or the transmission of data for external processing via communication protocols. The adopted approach stands out for its high precision and efficient application of mathematical techniques in converting raw intensity data into relevant thermal values, optimizing analysis and decision-making in advanced thermal monitoring systems.

### 2.8. PID Controller

The control of the thermal scanning system is based on the discrete analysis of the temperature matrix obtained from the thermal image. This analysis is used to either increase or decrease the laser power in order to maintain a target temperature within the treatment area.

The controller implemented in this system was a PID (Proportional–Integral–Derivative) controller, which is widely recognized for its effective balance between simplicity and performance [30]. This type of controller utilizes three control actions (proportional, integral, and derivative) to monitor and reduce the system error, which, in this case, is defined as the difference between the target temperature matrix and the temperature matrix in the region of interest corresponding to the tumor area.

Equation (7) describes the mathematical expression of the PID controller:

(7) $$u\left(t\right)=K_{p}e\left(t\right)+K_{i}\int_{0}^{t}e\left(t\right)dt+K_{d}\frac{de\left(t\right)}{dt}$$

where $K_{p}$, $K_{i}$, and $K_{d}$ represent the proportional, integral, and derivative gains, respectively. These constants must be properly tuned to optimize the system’s performance.

Since the temperature matrix feedback is obtained in discrete intervals of five seconds, Equation (7) was reformulated into its discrete time version, shown in Equation (8), where the integral and derivative terms are replaced by their discrete equivalents. The variable *T* denotes the sampling period.

(8) $$u\left(n\right)=K_{p}e\left(n\right)+K_{i}T\overset{n}{\underset{k=0}{\sum }}e\left(k\right)+K_{d}\frac{e\left(n\right)-e\left(n-1\right)}{T}$$

The gain parameters must be adjusted to improve the performance of the system. There are several ways to optimize these parameters, such as using the Ziegler–Nichols method, the Cohen–Coon method, genetic algorithms, or even neural networks [30]. Some of these methods require prior knowledge about the system and its responses, which can be difficult to obtain. Therefore, a trial-and-error method was chosen as a first approach for this proof of concept, allowing a more direct and specific tuning by iteratively adjusting the parameters based on the observations made. In the following section, this process is illustrated for the specific setup considered and within the overall integration of all sub-systems.

## 3. Results

### 3.1. Image Resizing

To test the system, the experimental setup shown in Figure 3 was used, with the black modeling clay selected as the target material for irradiation. After positioning the clay, the system was calibrated by placing the target at the correct distance from the mirrors and aligning the camera, as previously illustrated in Figure 9.

**Figure 16:**
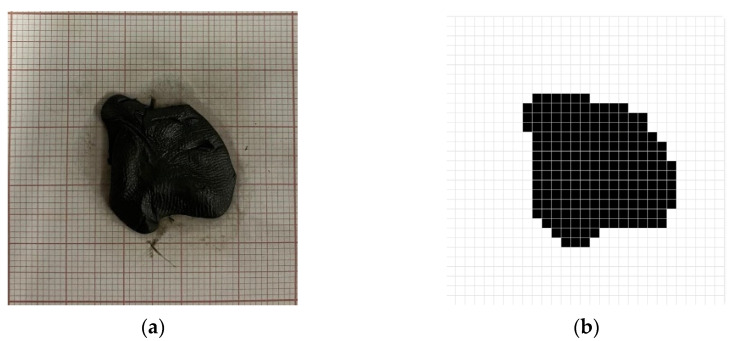
(**a**) Study object positioned at the center of the target and corresponding (**b**) digital image after image processing.

Next, an image of the region of interest was acquired and subjected to a processing pipeline that included segmentation, keystone effect correction, and resizing to a 30-row by 30-column matrix. This resizing step is crucial and was performed using bilinear or bicubic interpolation to ensure spatial fidelity was preserved and distortions were minimized. Figure 16 shows the result obtained after this processing. The resulting matrix was then used by the system to compute the parameters required for accurate mirror positioning.

### 3.2. Monitoring Temperature

To enable temperature control over time, thermal images were also periodically acquired and processed (Figure 15), undergoing an image processing pipeline similar to the one previously described.

In addition to image acquisition, the scanning speed, the target temperature, and the irradiation time were also defined through the user interface.

For the scan speed, a Δt of 1 ms was chosen. This value was determined by analyzing the diagnostic signals available on the servo amplifier boards to ensure system stability. The target temperature was set to 42 °C, consistent with the range used in low-temperature PTT [8]. A constraint was imposed so that the system could not exceed an overshoot beyond 46 °C. This limit was defined to avoid potential thermal damage to the phantom (and, in the intended application, to the irradiated cells).

**Figure 17:**
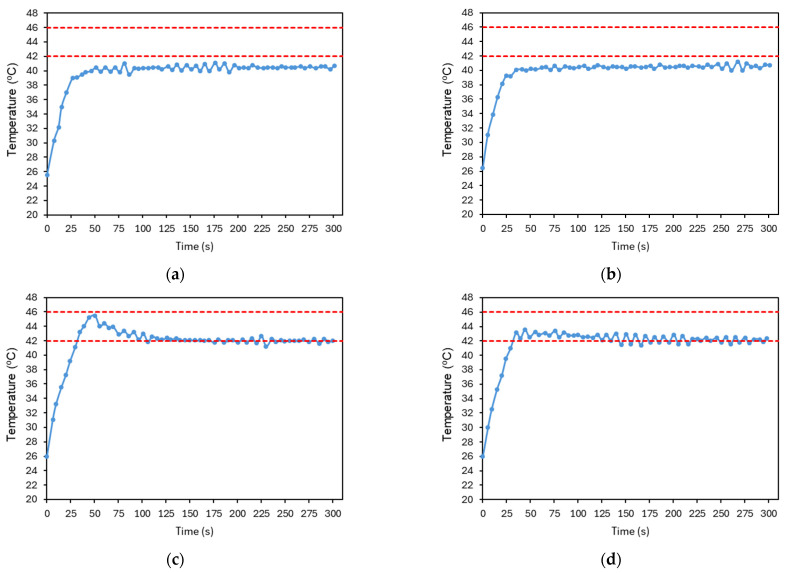
Resulting plots from the PID controller tuning process. (**a**) $K_{p}=25$, $K_{i}=0$, $K_{d}=0$. (**b**) $K_{p}=25$, $K_{i}=0$, $K_{d}=10$. (**c**) $K_{p}=25$, $K_{i}=1$, $K_{d}=10$. (**d**) $K_{p}=25$, $K_{i}=0.5$, $K_{d}=10$. The red dashed lines represent the reference temperature and the limit temperature defined to avoid thermal damage to the irradiated cells. The plotted temperature corresponds to the average temperature of the image at each time point. The accuracy of the temperature measurements is ±2 °C.

The parameters $K_{p}$, $K_{i}$, and $K_{d}$ were then tuned to ensure appropriate temperature control. Figure 17 illustrates part of the PID tuning process, presenting an example of its main steps. Initially, only a proportional gain, $K_{p}$, was applied and gradually increased until the system response approached the reference value and began to oscillate (Figure 17a). Next, a derivative gain, $K_{d}$, was introduced to reduce oscillations, as shown in Figure 17b. The resulting signal appeared stable but presented a steady-state error, which could be eliminated by adding an integral gain. In Figure 17c, a relatively high integral gain, $K_{i}$, was applied, which led to an overshoot, although within the previously defined temperature limits, it was considered suboptimal. To mitigate this, the integral gain was reduced (Figure 17d).

**Figure 18:**
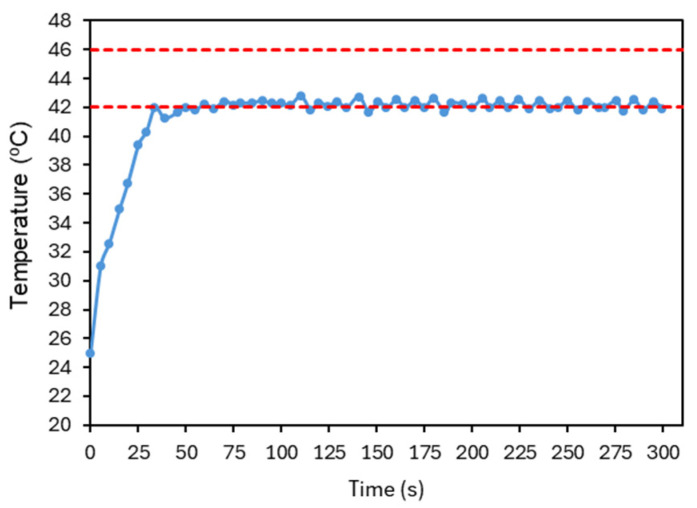
Final response of the average temperature of the image at each time point obtained after PID parameter optimization. $K_{p}=25$, $K_{i}=0.3$, $K_{d}=20$. The red dashed lines represent the reference temperature and the limit temperature defined to avoid thermal damage to the irradiated cells. The accuracy of the temperature measurements is ±2 °C.

**Figure 19:**
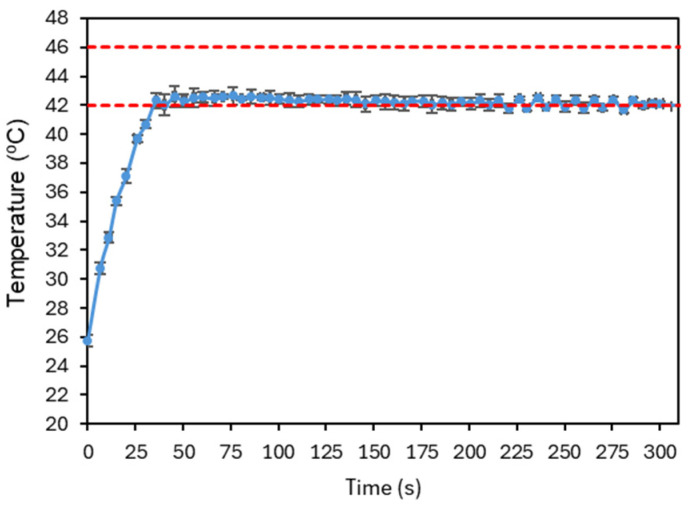
Plot illustrating a repeatability test under the conditions established after PID parameter optimization. Four experiments were considered, and the average temperature of the image at each time point, for each test, was averaged and the corresponding standard deviation is shown. The red dashed lines represent the reference temperature and the limit temperature defined to avoid thermal damage to the irradiated cells.

Figure 18 presents the final result of this tuning process. As some overshoot was still observable, the integral gain was further reduced and the derivative gain increased to suppress oscillations. The signal quickly converges to the reference temperature, where it stabilizes, with small fluctuations indicating that the controller continues to adjust the laser power over time. The system has shown good repeatability, as illustrated in Figure 19. The system was evaluated with four tests under the conditions established before. The number of repetitions was limited to reduce the probability of changing the conditions of the test.

These results were promising and suggest that this approach has potential for application in the intended system. However, several important considerations must be highlighted.

**Figure 20:**
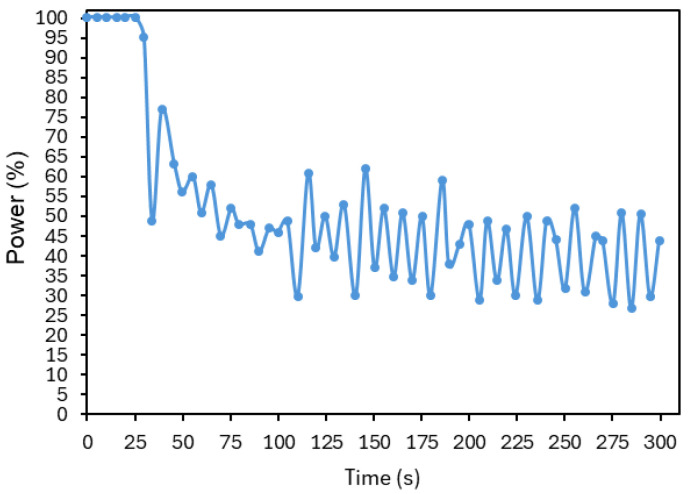
Laser power (in percentage) over time, corresponding to the controller illustrated in Figure 18.

First, it is desirable that the initial slope of the temperature rise be as steep as possible, as the goal is to reach the target temperature rapidly. As shown in Figure 20, which displays the laser power (in percentage) over time corresponding to the data shown in Figure 18, the system was already operating at its maximum power during this period. In this case, this slope could only be further increased by using a laser capable of delivering higher values of power.

Second, the stabilization of the target temperature depends on several factors, including the laser power, scanning speed, and the type of material for which the controller parameters were optimized. Thus, it is crucial to assess the system’s behavior in tumor cells, which exhibit additional dynamic characteristics, such as vascular activity.

Third, the fact that laser power varies significantly over time due to the controller’s action may present challenges in accurately determining the thermal dose. Therefore, exploring methods to stabilize this variation may also be of interest.

Lastly, this study focused on achieving homogeneous heating across the entire region of interest. However, in a therapeutic context, the ability to define different temperatures in multiple regions could be highly beneficial—for minimizing effects on healthy tissue and addressing tumor heterogeneity, among others. This would require a system with greater spatial modulation capability.

## 4. Discussion

The use of lasers as irradiation sources, and the interaction between laser beams and biological tissues, has been extensively studied over the years, particularly for heat generation. PTT has proven to be minimally invasive and highly selective, making it an excellent option for the treatment of superficial cancers, such as skin cancer.

Nevertheless, there remains a need to develop techniques that enable precise control of the irradiated area and its adaptation to the tumor size, aiming to minimize damage to surrounding healthy tissue. Using the PPT as motivation, a proof of concept based on an irradiation system using scanning optics was developed, allowing the control of energy, irradiation time, and target area through the use of galvanometric mirrors.

The operation of the system begins with the acquisition and processing of images from two cameras (visible and thermal). These images are utilized by the control unit to perform the scanning over the region of interest. The control unit consists of a Raspberry Pi 5, which communicates with a test board (BP-DAC61402EVM) equipped with a two-channel 12-bit DAC. This DAC generates analog control signals that, when fed to the mirror control boards, ensure the accurate positioning of the mirrors.

The control software was developed in Python and calculates and transmits the necessary parameters to generate the required analog signals, based on the initial planning of the irradiation area, target distance, and scanning times. A user interface, developed using Flask, provides a simple and intuitive interaction platform, allowing users to adjust certain DAC settings, define output voltages, and perform system calibration.

Following the design and assembly stages, the system was validated through an experimental trial using black modeling clay as the target material due to its high thermal absorption properties. The objectives were to verify the accuracy of the image processing, beam steering, and control functionalities, assess the suitability of the scanning pattern, and confirm the ability to monitor and control the temperature of the region of interest over time.

One of the main challenges in image processing was ensuring the proper alignment between the visible and thermal images. This issue stemmed from the parallax effect, caused by observing the target area from two different perspectives. A spatial adjustment based on the calculation of the centroids of both images was applied, minimizing distortions and information loss as much as possible. As seen in Figure 15, the thermal image does not perfectly match the visible image, a discrepancy attributed to the heat distribution within the study object.

Although the use of infrared thermometry offers advantages for non-contact, real-time temperature monitoring, it has known limitations [31]. These include potential inaccuracies due to emissivity variations, ambient light interference, dust, and surface reflections. Also, IR cameras can only detect surface temperatures and cannot assess sub-surface heating. Nevertheless, the proposed imaging methodology can be calibrated through extensive testing in different conditions and a correlation can be set between the different clinical parameters and the thermal camera information. In the future, combining this technique with others (e.g., photoacoustic thermometry to acquire in-depth information) can improve accuracy, while calibration and/or validation can be accomplished using invasive sensors (thermocouples or fiber-optic temperature sensors).

Regarding scanning times, a Δt of 1 ms was selected, as previously discussed, based on the analysis of diagnostic signals from the servo amplifier boards. It was also verified that the scanning time is constrained by the target distance and the chosen scanning pattern, as the system must ensure timely horizontal beam return.

Finally, the ability of the PID controller to regulate the temperature within the region of interest was evaluated. After optimization, the following parameters were obtained: $K_{p}=25$, $K_{i}=0.3$, and $K_{d}=20$. The resulting temperature response showed a rapid convergence to the setpoint, achieving stabilization without overshoot, thereby demonstrating the effectiveness and potential of the developed approach. Repeatability was tested and showed better uncertainty than that due to the accuracy of the camera. The uncertainty bars at Figure 19 are always lower that ±1 °C. However, the number of repetitions was limited to four per sample due to the need to maintain stable experimental conditions, which was not possible to guarantee with the present setup and phantom. The latter is particularly relevant since its properties (and even shape) will degrade/change after repeated irradiation/heating.

Despite the positive results, there are several optimizations and improvements of the developed system that are required before it can be considered ready for clinical applications. Future work should focus on the automation of certain processes, such as the calibration of the camera and the scanning system, to reduce positioning errors and enhance beam precision.

In addition, the integration of artificial intelligence techniques for real-time detection and processing of the region of interest would be highly beneficial, bringing the system closer to a realistic clinical application with patients. It will be equally beneficial to evaluate the use of models such as the Cumulative Equivalent Minutes at 43 °C (CEM43) or the Arrhenius damage model [32,33] to estimate the thermal damage of the tissues.

Concerning the control aspects, new scanning patterns should be investigated to further optimize scanning speeds. In particular, the use of triangular wave signals enables a scanning pattern similar to raster scanning, but with the beam scanning in both directions, thus increasing efficiency by eliminating the horizontal retrace phase [34].

Also, black modeling clay was used as the target material, but its thermal and optical properties differ significantly from those of real skin tumor tissues (with AuNPs, mediating the heat transfer). This will have an impact on the performance of the system, not only on the therapeutic process (due to the different absorption and photothermal conversion efficiencies) but also on the camera’s ability to capture information on the tumor’s region (dependent on the contrast with the healthy tissue) and thermal control over the region of interest during irradiation. Thus, to further demonstrate the performance of the system in real tumoral tissues and verify its clinical applicability, proper testing setups and methodologies (including the development of specific and more realistic phantoms) should be implemented. The overall system’s repeatability and accuracy will then be properly evaluated with further statistical characterization (e.g., larger sample sizes, thermal uncertainty estimation).

Finally, it will be essential to proceed to in vivo testing to evaluate the performance of the developed controller when applied to tumor cells, which present additional challenges due to their dynamic biological properties affecting heat dissipation. Moreover, the possibility of implementing a more customized temperature control across different areas within the region of interest should also be explored.

## 5. Conclusions

In this work, a proof of concept of a novel irradiation strategy was developed, aiming for future support of the photothermal therapy of superficial tumors, particularly skin cancer. The proposed system integrates two cameras—one in the visible spectrum and the other in the infrared—with a scanning module based on dual galvanometric mirrors. As far as the authors know, this is the first time such an integrated system, combining galvanometric scanning and thermal/visible imaging is presented, aiming for a future application such as PTT of a superficial cancer like skin cancer. The control software processes the visible camera input to identify the contour of the target (a phantom representing a skin tumor) and defines the scanning area accordingly. Using a raster scan pattern, the system dynamically drives the galvanometric mirrors to follow the defined region of interest. Simultaneously, data from the infrared camera enables real-time thermal monitoring, allowing the software to modulate the laser power to reach and maintain a user-defined target temperature for a specified duration.

The results demonstrated the feasibility and precision of the proposed approach, showing effective spatial and thermal control over the irradiated area. Although the system was applied only to a basic phantom, this allowed us to prove the effectiveness of the overall sub-systems and its capability to evolve under the overall requirements: define the region of interest, scan it, monitor the temperature increment, and provide feedback to the laser power to maintain a user selected temperature for the defined irradiation time. All the operational parameters of the system can be changed accordingly with the phantom being considered and the user requirements. These outcomes establish a strong foundation for future developments, including in vitro and in vivo validation, application of more robust and physiologically relevant approaches to estimate thermal damage, and advanced thermal modulation techniques for heterogeneous treatment zones. Thus, this system represents a promising step toward safer and more efficient photothermal therapy applications in clinical settings.

## Data Availability

The raw data supporting the conclusions of this article will be made available by the authors on request.

## Abbreviations

The following abbreviations are used in this manuscript:

ACS	American Cancer Society
WHO	World Health Organization
BCC	Basal Cell Carcinoma
SCC	Squamous Cell Carcinoma
UV	Ultraviolet
PTT	Photothermal Therapy
NIR	Near-infrared Radiation
AuNPs	Gold Nanoparticles
DAC	Digital-to-analog Converter
Rpi	Raspberry Pi
SPI	Serial Peripheral Interface
LWIR	Long-wave Infrared Radiation
JSON	JavaScript Object Notation
PID	Proportional–Integral–Derivative
CEM43	Cumulative Equivalent Minutes at 43 °C
